# Editorial: Cerebrospinal fluid biomarkers in psychiatric and neurological disorders

**DOI:** 10.3389/fpsyt.2026.1783064

**Published:** 2026-02-10

**Authors:** Kelly Garcia-Bohorquez, Jurjen Luykx, Matthew B. Johnson, Juan A. Gallego

**Affiliations:** 1 Northwell, New Hyde Park, NY, United States; 2 Department of Psychiatry at Donald and Barbara Zucker School of Medicine at Hofstra/Northwell, Hempstead, NY, United States; 3 Department of Psychiatry, Amsterdam University Medical Center, Amsterdam, Netherlands; 4 GGZ inGeest Mental Health Care, Amsterdam, Netherlands; 5 Amsterdam Neuroscience and Public Health Research Institutes, Amsterdam, Netherlands; 6 Department of Psychiatry and Neuropsychology, School for Mental Health and Neuroscience, Maastricht University Medical Center, Maastricht, Netherlands; 7 Stanley Center for Psychiatric Research, Broad Institute, Cambridge, MA, United States

**Keywords:** autoimmune diseases, biomarker, cerebrospinal fluid, delirium, depression, inflammation

Psychiatric and neurological disorders significantly contribute to morbidity, mortality, and disability (1, 2). These conditions present challenges in clinical practice due to their broad symptom spectrum and limited objective, specific diagnostic and follow-up tools, particularly in psychiatry.

To address this critical need, biomarker studies have emerged as a promising avenue for improving diagnostic, prognostic, and treatment approaches. The FDA-NIH Biomarker Working Group defines biomarkers as “a characteristic that is measured as an indicator of normal biological processes, pathogenic processes, or responses to an exposure or intervention” (3). Research exploring biomarkers includes neuroimaging, genetic, and molecular markers.

As psychiatric and neurological disorders primarily affect the brain, cerebrospinal fluid (CSF) is the biofluid of choice for biomarker measurement due to its free exchange of molecules with the brain. However, most studies traditionally focus on peripheral blood due to practical considerations.

The goal of this Research Topic was to compile CSF studies advancing understanding of psychiatric and neurological disorders and informing new treatments. Below, we briefly overview each study’s key findings and highlight future directions.

Wang et al. investigated the relationship between CSF levels of total ferritin, ferritin light chain, and ferritin heavy chain and depressive symptoms in 163 cognitively unimpaired participants and 380 individuals with mild cognitive impairment from the Alzheimer’s Disease Neuroimaging Initiative (ADNI) study. Three distinct trajectories of depressive symptoms were identified using Geriatric Depression Scale-15 (GDS-15) scores: (1) consistently low, (2) moderately increasing, and (3) rapidly increasing. They found that lower total CSF ferritin levels were associated with a rapidly increasing symptom trajectory. These findings are consistent with previous studies (4) and underscore the importance of considering iron metabolism in depression pathophysiology and its potential as a prognostic biomarker.

Piel et al. in a retrospective study investigating delirium biomarkers, analyzed CSF and blood samples from 16 patients with delirium, 33 patients with Alzheimer’s disease (AD), and 22 age-matched individuals without AD. Biomarkers related to neuroaxonal damage (NFL, UCHL-1, and tau protein) and neuroinflammation (GFAP) were measured in serum, while sTREM2 (neuroinflammation), SNAP-25, and NPTX2 (synaptic dysfunction) were measured in CSF. The authors found that serum NFL was elevated in patients with delirium compared to the other two groups. They also observed elevated tau protein in delirium patients compared to non-AD controls, suggesting that tau elevations are not exclusively indicative of dementia. In CSF, no significant findings were observed in delirium patients; however, elevated SNAP-25 levels were found in AD participants compared to non-AD controls, congruent with previous reports (5).

In another retrospective study, Seeliger et al. investigated blood-CSF barrier dysfunction in 419 subjects with immune-mediated neuropathies by comparing total CSF protein values to the albumin quotient (Qalb), calculated as the ratio of CSF albumin/serum albumin. They identified a significant divergence between CSF total protein and Qalb in nearly 9% of cases among patients with immune-mediated neuropathies. Notably, they observed that 2.3% of patients had a normal CSF total protein value despite an elevated Qalb, suggesting blood-CSF barrier dysfunction even in the presence of normal CSF total protein levels. These results highlight the importance of incorporating Qalb in the assessment of blood-CSF barrier dysfunction in immune-mediated neuropathies, rather than relying solely on CSF total protein values.

Finally, Hansen et al. measured several CSF biomarkers in 20 individuals with possible psychiatric autoimmune encephalitis (pAE), 7 individuals with definite pAE, and 27 individuals with AD. They found that P-tau 181 and total tau protein were significantly increased in individuals with AD and possible pAE, but not in those with definite pAE. The authors concluded that neuroaxonal cell damage may occur in possible but not in definite pAE and speculated that tau pathology in pAE might be a non-specific phenomenon occurring in the presence of specific antibody subclasses (such as IgLON5, glycine, titin, and recoverin autoantibodies) and non-specific neuropil autoantibodies. However, these results should be interpreted with caution due to the modest sample size.

This Research Topic offers insights into biological abnormalities in depression, delirium, immune-mediated neuropathies, and autoimmune encephalitis, with all studies reporting case-control differences or symptom severity associations. Replication studies are needed to generalize findings across broader patient populations and diverse geographical contexts. Multisite studies, like those by in Germany and Wang et al. using ADNI data, are essential for larger samples, increasing power and detecting meaningful clinical differences.

Future studies need mechanistic designs integrating CSF biomarkers with multimodal data: neuroimaging, genomics, proteomics, detailed clinical phenotyping. This approach is essential to characterize and disentangle biomarker alterations, identifying if they reflect primary disease mechanisms, downstream neurodegeneration/inflammation consequences, or state-related effects. Harmonized protocols and the application of biology and machine-learning systems could identify biomarker signatures, accelerating translation toward informed diagnostics and targeted interventions.

A common limitation in CSF research is its cross-sectional nature, partly due to the invasiveness of lumbar puncture that makes patients less inclined to participate in follow-up procedures. However, initiatives like ADNI (6) and the Psychiatric Biomarkers Network (PBN) (7) show feasibility for longitudinal studies with repeated lumbar punctures, especially with stakeholder engagement (8). Longitudinal designs are crucial for understanding disease mechanisms, determining causality, and assessing biomarker changes in relation to illness stages, symptomatology, or treatment response. Experimental/quasi-experimental approaches like treatment-response studies or Mendelian randomization may further clarify causal pathways.

Lastly, few submissions addressed CSF biomarker studies in common psychiatric disorders. Fluid biomarkers are radically transforming clinical development and treatment of neurodegenerative conditions like Alzheimer’s and Amyotrophic Lateral Sclerosis (9). A badly needed revolution in psychiatry requires similar efforts, leveraging lessons from neurological biomarkers and new tools for proteomics and data analysis to accelerate timelines.

In conclusion, this Research Topic underscores both the relevance and the untapped potential of the evolving field of CSF biomarker research in psychiatric and neurological disorders. It also teaches us what the shortcomings in the common designs are. Mechanistic, longitudinal and multimodal studies may in the future deepen our understanding of these pathologies and may ultimately translate into tangible benefits for patients through the development of precision diagnostics and targeted therapeutics.
